# Prescribing and using self-injectable antiretrovirals: How concordant are physician and patient perspectives?

**DOI:** 10.1186/1742-6405-6-2

**Published:** 2009-02-05

**Authors:** Robert Horne, Colin Kovacs, Christine Katlama, Bonaventura Clotet, Carmina R Fumaz, Michael Youle, Ranjababu Kulasegaram, Martin Fisher, Calvin Cohen, Jihad Slim, Peter Shalit, Vanessa Cooper, Christos Tsoukas

**Affiliations:** 1 Centre for Behavioural Medicine, Department of Policy & Practice, The School of Pharmacy, University of London, London, UK; 2 Department of Medicine, University of Toronto, Maple Leaf Medical Clinic, Toronto, Ontario, Canada; 3 Service des Maladies Infectieuses et Tropicales, Hôpital Pitié-Salpêtrière, Paris, France; 4 Institut de Recerca de la SIDA-Caixa Foundation, Barcelona, Spain; 5 Lluita contra la SIDA Foundation, Barcelona, Spain; 6 Royal Free Centre for HIV Medicine, London, UK; 7 Department of Genito-urinary Medicine, Guy's & St. Thomas' National Health Service Foundation Trust, London, UK; 8 Department of HIV/Genitourinary Medicine, Brighton and Sussex University Hospitals, National Health Service Foundation Trust, Brighton, UK; 9 Community Research Initiative of New England, Boston, Massachusetts, USA; 10 Saint Michael's Medical Center, Newark, New Jersey, USA; 11 Swedish Medical Center, Seattle, Washington, USA; 12 Montreal General Hospital, Montreal, Quebec, Canada

## Abstract

**Background:**

The selection of agents for any treatment regimen is in part influenced by physician and patient attitudes. This study investigated attitudinal motivators and barriers to the use of self-injectable antiretroviral agents among physicians and patients and measured the degree of concordance between physician and patient perspectives.

**Methods:**

Attitudes toward prescribing and usage of self-injectable antiretroviral therapy (SIAT) were assessed by structured interview in 2 cohorts sampled from the European Union and the USA: 499 HIV-treating physicians and 603 treatment-experienced HIV-infected patients. Motivators and barriers to prescribing SIAT were identified from statistical analysis of the associations between physicians' ratings of enfuvirtide-based therapy compared to standard oral-based therapy and 2 indicators of enfuvirtide prescribing behavior. Patients' attitudes were assessed by their responses to a written profile of enfuvirtide and their ratings of the likelihood of accepting a treatment offer.

**Results:**

Both indicators of SIAT prescribing behavior were predicted by the same pattern of physician beliefs. Nonprescribing was associated with: (1) the belief that offering enfuvirtide would be perceived negatively by patients, leading to treatment refusal and nonadherence; (2) the belief that prescribing enfuvirtide is harder to justify in terms of time/resources; and (3) a lack of confidence in the efficacy and use of enfuvirtide in practice (all p < 0.05). However, physicians' beliefs were not in concordance with patients' views. After reading a profile of enfuvirtide, 76% patients said that they would be moderately or highly likely to accept a treatment offer, although most (72%) had not discussed enfuvirtide with their doctor. Patients' beliefs predicted the likelihood of accepting enfuvirtide.

**Conclusion:**

Physician and patient beliefs about SIAT influence prescribing behavior and compliance yet may not be concordant, with patients having more positive attitudes towards SIAT than anticipated by physicians.

## Background

Although oral administration remains the most prevalent method of administering pharmaceutical agents, an increasing number of individuals depend on self-injectable medications for the effective treatment of their chronic illnesses and conditions including diabetes, hepatitis C, growth hormone deficiency, infertility, multiple sclerosis, and HIV. Chronic self-injection of medicinal therapeutics is associated with a number of potential drawbacks that can influence acceptability, including fear of needles, increased risk of disease disclosure, inconvenience, safety issues (including sharps disposal), local ISRs, and in many cases, the need for product reconstitution prior to injection. These novel challenges for physicians and patients can delay or prevent initiation of therapy – a phenomenon previously reported for injectable insulin therapy prescribed for patients with type 2 diabetes [1,2].

These issues are now salient in HIV therapy. Enfuvirtide is the first approved HIV-fusion inhibitor and the first approved antiretroviral (ARV) agent that cannot be administered orally. Currently, it is formulated as a powder to be reconstituted and injected subcutaneously. Although enfuvirtide has demonstrated activity against HIV-1 strains that are resistant to all 3 of the original ARV drug classes [3], has a proven efficacy and safety profile [4-7], and is cited by major HIV-treatment guidelines for use in treatment-experienced patients [8-11], research suggests that it is under-utilized in current clinical practice [12].

The translation of novel health technologies, such as enfuvirtide, into health gain for affected individuals is dependent on the behavior of clinicians and patients. The clinician needs to make an appropriate treatment recommendation and the patient must be committed to adhere to the appropriately prescribed treatment. Physician and patient behavior and decisions about treatment are likely to be influenced by both perceptual factors (eg, beliefs, attitudes, and preferences) and practical factors (eg, capacity and resources), as has been shown previously for the management of heart failure [13].

Facilitating optimal patient management requires an understanding of both the physician's and the patient's perspectives of treatment and, in this situation, the challenges of prescribing and adhering to ARVs. Research into patient adherence to ARVs has improved our understanding of patient perspectives and has identified some of the key factors influencing patient motivation and the ability to initiate and to continue with treatment [14-16]. However, there are no recent studies that have considered physician perspectives on ARVs and thus we know little about the perceptual and practical barriers to the optimal prescribing of these drugs. The relatively low uptake of SIAT, despite the available evidence supporting its efficacy and safety, suggests that there is a need for a systematic study of the barriers against and drivers for the use of such therapies from both physician and patient perspectives.

Previous pilot qualitative research suggests there may be a range of barriers influencing enfuvirtide prescribing and uptake/acceptance and that some potentially important disconnects may exist between physician and patient perceptions of self-injectable treatment [17]. In order to identify, quantify, and compare physicians' and patients' beliefs about self-injectable treatment and to examine the relationship between their beliefs and SIAT use in clinical practice, we conducted a large-scale empirical investigation of physician and patient perceptions self-injectable treatment, using enfuvirtide as the treatment example.

The principal aims of our study were to understand the underlying motivations for physicians to prescribe or not to prescribe self-injectable treatment; to understand the motivation behind treatment-experienced patients' acceptance of enfuvirtide as a treatment; and to identify potential incongruence between the perceptions of physicians and their patients regarding self-injectable treatment in HIV. This research should be useful in developing evidence-based interventions to align treatment motivations for both physicians and patients, which could have broader applications in other therapeutic areas where self-injection is needed.

## Methods

Beliefs about SIAT were assessed by questionnaires administered in structured interviews of physician and patient cohorts in Germany, France, Italy, Spain, the UK, and the USA between May and August 2005. The study was comprised of 3 phases: (1) identification of potential barriers and drivers to prescribing SIAT among a physician cohort; (2) evaluation of attitudes, in a patient cohort, toward enfuvirtide (using a written profile of the drug as a basis), including patient willingness to accept a treatment offer of enfuvirtide from their physician; and (3) a qualitative comparison between the perceptions identified for physician and patient cohorts. The questionnaires were designed by the study group of researchers and clinicians on the basis of the results of an exploratory, qualitative study [17]. Questionnaires were piloted in the UK and then translated to the required languages. Participants were reimbursed according to local guidelines and good practice. All interviews were conducted by trained medical interviewers and in accordance with the Data Protection Act. Each participant was assured complete confidentiality.

### Physician cohort

#### Enrolment procedure

Physicians at HIV centers and hospitals geographically distributed across each country were initially approached via telephone and screened for eligibility. Physicians were eligible for the study if they were HIV- or infectious disease-specialists treating patients with HIV, had at least 3 years of experience prescribing ARVs, and claimed a minimum of 15% treatment-experienced patients within their clinic. (In this study 'treatment-experienced patients' were defined as patients who had been exposed to [but had not necessarily failed therapy on] at least 8 different ARVs, including those in their current regimen. These patients are generally suitable candidates for enfuvirtide treatment at their next treatment change, according to authoritative HIV-treatment guidelines [8-11].)

##### Assessing physician attitudes to enfuvirtide and prescribing behavior

Physician beliefs about enfuvirtide were assessed on the basis of their responses to 31 statements about an enfuvirtide-based regimen relative to a standard oral ARV-based regimen. The belief statements represented potential barriers against and drivers for the use of enfuvirtide for treatment-experienced patients that had been previously identified in a qualitative study [17]. Physicians rated their level of agreement with each of the belief statements on a 7-point Likert-scale (where 1 = strongly disagree, 7 = strongly agree, and 4 = neutral). Responses were subjected to principal components analysis (PCA), transforming group beliefs into core themes or factors.

### Physician prescribing behavior

#### 1. Physicians' reports of their current enfuvirtide prescribing levels

On the basis of their responses, physicians were classified into 3 different prescriber categories: 'nonprescribers': 0 patients prescribed enfuvirtide; 'lower prescribers': 1–4 patients prescribed enfuvirtide; and 'higher prescribers': = 5 patients prescribed enfuvirtide.

#### 2. Prescribing intentions in case scenarios

Physicians evaluated 2 hypothetical patient case scenarios – one of a former IVD user and the other of a patient with a history of nonadherence. The patient-case scenarios generated by an international expert HIV physician panel were to include clear candidates for enfuvirtide, as indicated by international guidelines [8-10]. Physicians were asked to choose between 2 treatment options, one of which included enfuvirtide (Table 1). A similar approach has been used in previous studies investigating physician prescribing behavior [13].

**Table 1:** Summary of case scenarios

**Two scenarios were used to evaluated physicians' prescribing intent for patients who are clear candidates for receiving a new ARV class to achieve treatment goals as recommended by international guidelines**
**Case 1: Ex-IVD user**
• 46-Year-old white heterosexual male infected with HIV through IVD use and diagnosed in 1989
• Peak VL 100 000 copies/mL, CD4 nadir 40 cells/mm^3^; current VL 20 000 copies/mL, CD4 145 cells/mm^3 ^(declined 30 cells/mm^3 ^since previous visit)
• Treatment history includes ZDV, d4T, ddI, ddC, EFV, NVP, SQV, IDV, SQV/r, NFV, LPV/r, and IDV/r (severe reactions with NNRTIs and developed K103N mutation; gastrointestinal distress with many PIs, particularly LPV/r; patient also suffers from severe lipodystrophy)

Two regimen options recommended by HIV physician panel:
• **Option X**: TPV/r + enfuvirtide + 3TC + TDF
• **Option P**: ATV/r/SQV + 3TC + TDF

**Case 2: Patient with history of nonadherence**
• 42-Year-old black African female diagnosed with AIDS (pneumocystis pneumonia) in 2002
• At diagnosis, VL 100 000 copies/mL and CD4 40 cells/mm^3^; current VL 2000 copies/mL and CD4 200 cells/mm^3 ^(210 cells/mm^3 ^at previous visit)
• Treatment history includes ZDV, ABC, TDF, 3TC, FTC, EFV, ATV/r, and LPV/r
• Resistance profile includes 41L, 215Y, 184V, and 103N
• Nonadherence associated with gastrointestinal side effects, failure to take drugs on days that patient feels healthy and running out of drugs during an extended trip to Africa

Two regimen options recommended by HIV physician panel:
• **Option X**: TPV/r + enfuvirtide + 3TC + TDF
• **Option P**: FPV/r/SQV + 3TC + TDF

### Patient cohort

The patient enrolment procedure was approved by the European Community Advisory Board, a recognized international AIDS patient organization and one the 3 working groups of the European AIDS Treatment Group. Eligible patients were at least 16 years of age, treatment experienced (according to the study definition), and currently receiving ARV therapy. Patients were primarily identified by individuals enrolled in the physician cohort, who were asked to establish whether the next 2 treatment-experienced patients seen would be willing to participate in a telephone research interview. If a patient refused, physicians were asked to approach subsequent treatment-experienced patients until 2 candidates had been identified. Additional patients were recruited through nonstudy physicians, nurses, patient organizations, and, in the UK and Spain, through posters advertising the study in general practitioners' offices and patient organizations (using a 'freephone' contact number). Standard consent procedures were followed.

#### Assessing patient attitudes toward SIAT

Structured interviews (approximately 30 minutes) were conducted by telephone (except in Italy where, due to data protection laws, the interviews were conducted face-to-face). Patients rated their level of agreement with a series of 29 attitudinal statements representing potential barriers and motivators to using SIAT that were derived from previous qualitative research [17]. Responses were scored on a 7-point Likert-style scale, where 1 = 'strongly disagree', 7 = 'strongly agree', and 4 = neutral. Participants were shown a written profile describing enfuvirtide (available on request from the authors) and were asked a range of questions regarding the product's level of appeal and acceptability. Those not currently taking enfuvirtide were asked to rate on a 7-point scale (where 1 indicated 'not likely at all' and 7 indicated 'very likely') their likelihood of accepting this drug. Responses were used to group patients into 3 categories: unlikely to accept (responses between 1–3); moderately likely to accept (responses of 4); or likely to accept (responses between 5–7).

### Statistical methods

Target sample sizes were chosen to be representative of each country. The overall sample size was selected to allow for 90% confidence that error would be within ± 10%. Our overall target was 510 physicians (75 physicians each from France, Italy, Spain, and the UK, 60 from Germany, and 150 from the USA) and 650 patients (100 patients each from France, Germany, Italy, Spain, and the UK, and 150 from the USA).

Data were analyzed using Statistical Package for the Social Sciences (SPSS^® ^12.0, SPSS Inc, Chicago, Illinois) software. Associations between physician characteristics and prescriber category were explored using the chi-square test for categorical variables and 1-way ANOVA with Tukey's post-hoc comparison tests for continuous variables.

Principal components analysis (PCA) with varimax rotation was used to identify the main themes (factors) underpinning physicians' and patients' attitudes to SIAT. Items with low values on the Keiser Meyer Olkin (KMO) measure of sampling adequacy were excluded. The internal reliability of the resulting measure of each theme was assessed using Cronbach's alpha. A value of ≥ 0.6 was considered acceptable for this exploratory study.

Logistic regression was used to calculate odds ratios and 95% confidence intervals for the relationship between physician beliefs and the likelihood of prescribing a SIAT (according to current enfuvirtide prescribing rates and choice of regimen in the case scenarios), controlling for demographic characteristics (number of HIV patients managed and country). Continuous predictor variables were dichotomized at the median value to aid interpretation of the odds ratio.

One-way ANOVA, with Tukey's post-hoc comparison tests was used to explore the association between a patient's beliefs and their stated likelihood of accepting an offer of enfuvirtide from their physician. A p value of < 0.05, using a 2-tailed analysis, was considered significant.

## Results

### Physician cohort

#### Participants

Of 948 physicians approached, 191 did not meet eligibility criteria and 258 study-eligible physicians declined to participate, resulting in a final sample size of 499 physicians. Table 2 summarizes their demographic and practice characteristics.

**Table 2:** Demographics and practice characteristics of physician sample; n (%) unless otherwise stated

	**Nonprescriber**^**a**^ **(0 patients)**	**Lower prescriber**^**a**^ **(1–4 patients)**	**Higher prescriber**^**a**^ **(≥ 5 patients)**	**Total**
Physicians	120 (24)	204 (41)	175 (35)	499 (100)
Physicians with missing data (excluded from analysis)	8 (7)	2 (1)	1 (1)	11 (2)
Years practicing as an HIV-related physician, median (range)	12.4 (3–30)	11.8 (3–31)	11.5 3–30)	11.0 (3–31)
**Medical specialty**				
*HIV/AIDS specialist*	58 (52)	113 (56)	87 (50)	258 (53)
*ID specialist*	41 (37)	73 (36)	69 (40)	183 (38)
*Other*^*b*^	9 (8)	10 (5)	11 (6)	30 (6)
**Base**				
*Hospital*	88 (79)	140 (69)	123 (71)	351 (72)
*Office*	24 (21)	62 (31)	50 (29)	136 (28)
**Patients with HIV currently managed**				
*≤ 100*	49 (44)	36 (18)	11 (6)	96 (20)
*101–300*	57 (51)	137 (68)	112 (64)	306 (63)
*301+*	6 (5)	29 (14)	51 (29)	86 (18)
**Proportion of these patients who are treatment experienced**^**c**^				
*< 10%*^*d*^	4 (4)	8 (4)	1 (1)	13 (3)
*10% to 24%*	49 (44)	93 (46)	62 (36)	204 (42)
*25% to 49%*	41 (37)	60 (30)	74 (43)	175 (36)
*50% to 74%*	13 (12)	33 (16)	28 (16)	74 (15)
*≥ 75%*	5 (5)	8 (4)	9 (5)	22 (4)
**Country**				
*UK*	31 (28)	23 (11)	7 (4)	61^e ^(13)
*France*	9 (8)	38 (19)	28 (16)	75 (15)
*Germany*	10 (9)	17 (8)	31 (18)	58 (12)
*Spain*	14 (13)	39 (19)	21 (12)	74 (15)
*Italy*	18 (16)	25 (12)	32 (18)	75 (15)
*USA*	30 (27)	60 (30)	55 (31)	145 (30)

Percentages may not total 100 because of rounding.

^a^Based on the number of patients currently prescribed enfuvirtide.

^b^GP/FP/IM specialist/PCP.

^c^Defined as patients who had been exposed to (but who had not necessarily failed therapy with) at least 8 different ARVs, including those in their current regimen.

^d^At screening, these physicians stated that they had ≥ 15% treatment-experienced patients within their clinic and so were eligible to participate in the study.

^e^In the course of fieldwork it was decided by the study team to reduce the number of physicians to be interviewed in the UK from 75 to 62, to represent a smaller physician population in this country.

#### Physician beliefs about enfuvirtide

PCA of physicians' responses to the 31 belief statements about an enfuvirtide-based vs a standard oral-based ARV regimen identified 7 factors or 'themes'. The individual belief statements loading on each of the 7 factors are available on request. The Cronbach's alpha values indicate that 6 of the 7 scales had an acceptable internal reliability.

#### Physician prescribing intentions and behaviour

##### 1. Enfuvirtide-prescribing rates

120 physicians (24%) were classified as 'nonprescribers', 204 (41%) were 'lower prescribers,' and 175 (35%) were 'higher prescribers' (Table 2). The proportion of physicians who were higher prescribers differed by country (χ^2 ^(5) 24.79, p < 0.001). Those based in Germany were more likely to be higher prescribers (χ^2 ^[1] 6.93, p < 0.01), while those based in the UK were less likely to be higher prescribers (χ^2 ^[1] 17.58, p < 0.001). Physicians who were categorized as 'higher prescribers' personally managed a greater number of patients with HIV (χ^2 ^[2] 54.81, p < 0.001) than did lower and nonprescribers of enfuvirtide. When physicians were asked to rate their experience of prescribing self-injectable therapy *for any condition*, there were significant differences between the 3 prescriber categories (F [2498] = 7.87, p < 0.001). Higher prescribers indicated a greater level of experience than both lower (p < 0.05) and nonprescribers (p < 0.001). Higher prescribers also were more likely to take part in clinical trials/research (χ^2 ^[2] = 17.58, p < 0.001) and author papers about the management of HIV (χ^2 ^[2] = 9.20, p < 0.005) than were lower and nonprescribers.

#### Physician beliefs associated with enfuvirtide-prescribing intentions and behavior

Table 3 shows the multivariate odds ratios (95% confidence intervals) for physician beliefs influencing enfuvirtide-prescribing intention and behavior. A similar pattern of physician beliefs about SIAT predicted both prescribing indicators. Four attitudinal factors were particularly influential in terms of enfuvirtide prescribing.

**Table 3:** Multivariate odds ratios (95% confidence intervals) for physicians' beliefs about enfuvirtide regimens compared with standard oral-based regimens for treatment-experienced patients^a^

**Physician belief themes**	**Enfuvirtide-prescriber category**		**Case scenarios**	
	'Higher' (≥ 5 patients currently prescribed)^b^ (n = 175)	'Non' (no patients currently prescribed)^c^ (n = 120)	Physicians selecting enfuvirtide option	
			Case 1^d^ (n = 323)	Case 2^e^ (n = 214)
Personal confidence in the efficacy of enfuvirtide and its use in practice	3.35^¶ ^ (2.20, 5.08)	0.32^¶ ^ (0.19, 0.53)	2.44^¶ ^ (1.62, 3.66)	2.10^‡ ^ (1.44, 3.07)
Enfuvirtide is associated with treatment refusal and nonadherence	NS	2.05^‡ ^ (1.29, 3.25)	0.59^† ^ (0.40, 0.86)	0.69* (0.47, 0.99)
Enfuvirtide is difficult to justify in terms of time and resources	0.63* (0.42, 0.94)	1.69* (1.07, 2.64)	0.57^‡ ^ (0.39, 0.83)	NS
An enfuvirtide treatment offer is likely to jeopardize patients' trust	0.56^‡ ^ (0.37, 0.83)	1.86^† ^ (1.19, 2.92)	NS	NS
Patients are likely to perceive that drawbacks of enfuvirtide outweigh its benefits	NS	NS	NS	NS
Concerns about the risks of self-injectable therapies	NS	NS	0.52^¶ ^ (0.37, 0.78)	NS
Enfuvirtide is more suitable for nonadherent/chaotic patients	NS	NS	NS	2.44^¶ ^ (1.67, 3.57)

^a^Controlling for number of patients with HIV managed by each physician and for country.

For the statistical comparisons:

^b^Compared with lower and nonprescribers.

^c^Compared with higher and lower prescribers.

^d^Compared with physicians who did not select the enfuvirtide option.

^e^Compared with physicians of patients who were currently prescribed enfuvirtide.

*p < 0.05; ^†^p < 0.01; ^‡^p < 0.005, ^¶^p < 0.001.

NS = no statistically significant difference between comparators.

Low rates of enfuvirtide prescribing in practice were associated with a:

- Lack of personal confidence in the efficacy of enfuvirtide and its use in practice

- Belief that enfuvirtide is associated with patient treatment-refusal and nonadherence

- Belief that an enfuvirtide prescription is difficult to justify in terms of time and resources

- Belief that a treatment offer of enfuvirtide is likely to jeopardize a patient's trust

##### 2. Prescribing intentions in the case scenarios

In case 1 (ex-IVD user), 66% of physicians overall chose to use enfuvirtide, while only 45% of physicians chose enfuvirtide for a patient with a history of nonadherence to ARVs (case 2). The beliefs associated with choosing enfuvirtide for each case scenario are shown in Table 3.

### Patient cohort

Of the 1314 patients approached, 273 did not meet eligibility criteria and 439 study-eligible patients declined to participate, resulting in a study cohort of 603 patients. Table 4 summarizes their demographic characteristics.

**Table 4:** Characteristics of interviewed patients (n = 603)

	**n (%)**
**Gender**	
Male	437 (72)
Female	166 (28)
**Age (years)**	
16–25	5 (1)
26–35	76 (13)
36–45	317 (53)
46–55	163 (27)
≥ 56	42 (7)

**Country**	
UK	50 (8)
France	100 (17)
Germany	101 (17)
Spain	101 (17)
Italy	101 (17)
USA	150 (25)

**Route of infection**	
Homosexual contact	212 (35)
Heterosexual contact	172 (29)
IVD use	138 (23)
Other	81 (13)

**Education**	
Some high school/secondary school	161 (27)
Graduated high school/secondary school	174 (29)
Some college training	146 (24)
Graduated college/university	77 (13)
Completed postgraduate degree	18 (3)
**Employment**	
Employed	218 (36)
Homemaker	17 (3)
Student	11 (2)
Retired	68 (11)
Unemployment benefit	148 (25)
Incapacity benefit	134 (22)

#### Current use of enfuvirtide

Of the 603 interviewed patients, 61 (10%) were currently receiving enfuvirtide. Of the 542 patients who were not currently receiving enfuvirtide (categorized as 'nonusers'), 150 (28%) had discussed SIAT with their physician, resulting in an offer of enfuvirtide therapy from their physician in 56 cases (37%). Among nonusers who had been offered enfuvirtide, 17/56 (30%) had initiated therapy, but subsequently discontinued treatment.

#### Likelihood of accepting enfuvirtide if offered by physician

After reviewing the description of enfuvirtide, 180 of the 516 patients who responded (35%) indicated that they would be likely to accept this product if offered to them by their physician (score 5–7), 214 (41%) would be moderately likely to accept (score = 4), and 122 (24%) would be unlikely to accept (score = 1–3).

#### Patients' beliefs about SIAT

The 3 most prevalent PCA-derived core beliefs were that their doctor had positive views about self-injectable therapies (52%), that SIAT would be effective and preferable to other treatment options (35%), and that there would be barriers to using SIAT (27%).

#### Predictors of likelihood of accepting enfuvirtide if offered by physician

Patients who indicated they were likely to accept therapy were more likely to believe that SIAT is effective and preferable to other treatment options (Table 5). In contrast, patients who indicated that they were unlikely to accept enfuvirtide were more likely to perceive barriers to adhering to SIAT, to have concerns about self-injecting, and resistance to their doctor recommending SIAT although, interestingly, the mean scores for these last 2 beliefs among this patient subset were below the midpoint, indicating modest overall disagreement with these beliefs.

**Table 5:** Beliefs influencing patients' likelihood of accepting enfuvirtide if offered to them by their physician

	**Likelihood of accepting enfuvirtide**		
	**Likely**^**a**^	**Moderately likely**^**b**^	**Unlikely**^**c**^
	**(n = 180)**	**(n = 214)**	**(n = 122)**
	**Mean (SD)**	**Mean (SD)**	**Mean (SD)**
Perception that enfuvirtide is more effective and preferable to oral therapy (F [2502] = 102.5, p < 0.001)	4.39 (1.26)	3.34 (1.08)	2.53 (0.91)
Perceived barriers to adherence (F [2503] = 77.26, *p *< 0.001)	2.45 (1.20)	3.52 (1.27)	4.27 (1.37)
Concerns about self injecting (F [2513] = 36.0, *p *< 0.001)	2.65 (1.14)*	3.29 (1.08)*	3.74 (1.18)
Resistance to doctor recommending self-injectable therapies (F [2513] = 72.2, p < 0.001)	1.90 (1.12)	2.95 (1.35)	3.76 (1.61)
Perception that doctors have positive views about enfuvirtide (F [2233] = 8.66, p < 0.001)	1.57 (0.50)	1.35 (0.48)	1.23 (0.42)

a, b, c: refer to the product profile (Figure 1) preference score. Patients responded to the question, "If you were offered this product by your physician, how likely would you be to accept it?" using ratings of ^a^1–3, ^b^4, and ^c^5–7 on a 7-point scale, where 1 is 'very likely', 4 is a neutral opinion, and 7 is 'not likely at all.'

All comparisons p < 0.001 except for *p < 0.005.

### Comparison of attitudes and beliefs between physician and patient cohorts

A number of interesting contrasts can be drawn from the findings of the physican and patient cohorts. Although 76% of physicians had greater confidence in the efficacy and clinical use of SIAT vs oral ARVs for treatment-experienced patients, only 35% of patients perceived that SIAT would be efficacious and preferable to other treatments. However, while 40% of physicians felt that patients' perceived drawbacks of enfuvirtide outweighed its benefits, fewer than 1 in 3 patients agreed with this opinion.

Further, nearly half of all interviewed physicians believed that enfuvirtide is associated with increased nonadherence and treatment refusal, whereas only 27% of patients agreed that there were perceived barriers to adherence with self-injectable therapy, and just 24% indicated they would resist enfuvirtide therapy if their physician recommended it. Finally, a small number of physicians (8%) reported that prescribing an enfuvirtide-based regimen would be more likely than an oral ARV-based regimen to jeopardize their patient's trust in them, whereas 15% of patients indicated that a strong recommendation of a SIAT by their doctor would damage the relationship between them.

## Discussion

In this large-scale empirical study we identified and quantified the experiences, perceptions, and beliefs of physicians and patients about SIAT, and examined the relationship between their beliefs and its acceptance and use in clinical practice. The findings suggest that, even when SIAT is likely to be optimal for therapeutic success, there are multiple physician and patient factors influencing whether a SIAT is prescribed to eligible patients, and whether eligible patients are likely to comply with a SIAT.

Our findings indicate that current use of enfuvirtide in the management of HIV infection is determined not only on the basis of scientific evidence but also by physician experiences and attitudes toward self-injectable therapy. Higher enfuvirtide prescribers had a more positive view about its efficacy, were more confident that prescribing SIAT would not jeopardize their patients' trust and were less likely to believe that prescribing SIAT would be difficult to justify in terms of time and resources. Differences in beliefs of this type also were apparent between physicians who selected the enfuvirtide option in the two case scenarios vs those who did not. The fact that our findings were consistent across the 2 prescribing outcomes further supports validity.

Physicians who managed a greater number of patients receiving enfuvirtide also managed more patients with HIV overall and had more experience with prescribing self-injectable therapies for other conditions. These findings corroborate those of other studies showing that a lack of physician experience with self-injectable therapy is a barrier to prescribing self-injected medications [18].

The patients we studied would generally benefit from and be eligible for an enfuvirtide-containing regimen at their next treatment change, according to HIV treatment guidelines in use at the time of data collection [8-11]. Interestingly, relatively few patients who currently were not taking enfuvirtide had even discussed this treatment option with their doctor. However, if patients had been stable and virologically suppressed on their current regimen (a parameter that we did not measure), then the perceived need for such a discussion would be low. After viewing the product profile for enfuvirtide, more than 75% of patients said they would be at least moderately likely to accept this therapy if offered to them by their physician. Patients who were likely to accept such an offer were more likely to believe that SIAT is efficacious and preferable to other treatment options, and were less in agreement with beliefs associated with perceived barriers to adhering to SIAT, concerns about self-injecting, and resistance to their doctor recommending self-injectable therapy.

Perhaps the most interesting aspect of this study was the finding that physicians specializing in HIV appeared to have somewhat different perceptions about SIAT than did treatment-experienced patients. Nearly half of all interviewed physicians felt that enfuvirtide was associated with increased nonadherence and treatment refusal, whereas only 27% of patients agreed with perceived barriers to adherence, and only 24% indicated they would resist this type of therapy if recommended by their physician. Our results suggest that patients' perceptions of SIAT may be more positive than previously thought and patients may be more receptive to initiating SIAT than currently believed by physicians who manage patients with HIV.

We acknowledge study limitations associated with self-reporting, self-presentation, and recall bias inherent in research of this nature. However, we attempted to minimize this potential bias by introducing our survey in a neutral manner with the assurance of complete confidentiality. Questions also were posed in a neutral manner, participants only had to agree/disagree with statements attributed to other physicians/patients, and a combination of both positive and negative statements was included.

For the physician cohort analysis, our classification of physicians as 'higher' vs 'lower' prescribers does not take into account the number of triple-class-experienced patients under their care, so may not accurately reflect their true propensity to prescribe enfuvirtide to eligible patients. It also does not take into account what resources, supportive of enfuvirtide prescribing, were available to individual physicians. It could be hypothesized that higher prescribers were, for example, supported by a broader multidisciplinary team of health care professionals than were lower prescribers or nonprescribers, thus permitting a higher level of support in encouraging patients to persist with injectable therapy.

Since subjects in the patient cohort were mainly referred to the study by their HIV clinician, our sample may be biased towards those with positive attitudes towards the medical profession or research. Further bias may have been introduced by the enfuvirtide profile, as some patients may not have fully understood its contents, thus compromising their ability to make informed choices. Finally, Cronbach's alphas were relatively small (all but one within the range 0.6–0.8). However, a value of 0.7 is generally thought to be an acceptable reliability coefficient, while 0.6 is considered acceptable for an exploratory study [19,20].

We did not collect information on viral load. Attitudes towards SIAT may differ between those who are suppressed on their current therapy and those for whom a change in therapy might be indicated. Research in other conditions suggests that barriers to self-injecting are lower among people who perceive their individual need for a specific treatment to be high [21].

Since this study was conducted, several new agents have been licensed for the treatment of ARV-experienced patients. These include new classes of drugs including CCR5-inhibitors and integrase inhibitors as well as a new potent protease inhibitor. As HIV physicians now have more options available for the treatment of ARV-experienced patients, the results of this study may now be applicable to a smaller group of treatment-experienced patients. However, the finding that treatment-experienced patients' attitudes towards SIAT may be more positive than their physicians anticipate suggests that discussions about treatment options should not exclude self-injectable treatments on the basis of presumptions about patients' negative attitudes towards them.

The findings of this study are comparable to those in other long-term conditions such as diabetes and oesteoporosis, where patients' views about self-injectable medications were important determinants of their treatment decisions [21-24]. They also support earlier research that suggests physicians who manage patients with HIV and treatment-experienced patients may have different perceptions about SIAT [17]. Certainly in other therapeutic areas, including asthma and oncology, physician-patient differences in the perception of self-injectable therapies have been identified as having a range of adverse implications, including a potential contribution to suboptimal patient care [25-27]. While we have no direct evidence that enfuvirtide is not being prescribed according to guidelines, other research certainly suggests that this is the case [12]. Understanding both patient and physician perceptions is a first step in designing evidence-based interventions to facilitate the optimum use of SIAT in treatment-experienced patients with HIV. Our findings suggest that more needs to be done to integrate evidence-based physician-oriented interventions within medical education programs to ensure that all physicians make informed treatment decisions in conjunction with their patients when prescribing a SIAT.

## Conclusion

Our data indicate that, for SIAT, treatment decisions are strongly linked to the personal perceptions and beliefs of both physicians and patients. The results of this study involving enfuvirtide provide an important insight into the psychology behind the use of self- injectable therapy and our findings potentially have implications across a range of therapeutic areas in which parenteral administration of new medicines may be optimal for selected patient populations. The relevance of SIAT is likely to grow given the number of parenterally administered antiretrovirals representing novel mechanistic classes that are currently in clinical development.

## Abbreviations

3TC: lamivudine; ABC: abacavir; AIDS: autoimmune disease syndrome; ANOVA: analysis of variance; ARV: antiretroviral; ATV/r: atazanavir/ritonavir; d4T: stavudine; ddC: zalcitabine; ddI: didanosine; EFV: efavirenz; FP: family practitioner; FPV/r: fosamprenavir/ritonavir; FTC: emtricitabine; GP: general practitioner; HIV: human immunodeficiency virus; ID: infectious disease; IDV: indinavir; IDV/r: indinavir/ritonavir; IM: internal medicine; ISRs: injection site reactions; IVD: intravenous drug; KMO: Keiser Meyer Olkin; LPV/r: lopinavir/ritonavir; NFV: nelfinavir; NNRTIs: nonnucleoside reverse transcriptase inhibitors; NVP: nevirapine; PCA: principal component analysis; PCP: primary care physician; PIs: protease inhibitors; SIAT: self-injectable antiretroviral therapy; SQV: saquinavir; SQV/r: saquinavir/ritonavir; TDF: tenofovir; TPV/r: tipranavir/ritonavir; VL: viral load; ZDV: zidovudine.

## Competing interests

RH has received unrestricted educational grants from and/or research consultancy for Abbott, Boehringer Ingelheim, Bristol-Myers Squibb, Gilead Sciences, GlaxoSmithKline, Pfizer, Roche and Merck. CKa has received reimbursements and study funding from Roche. BC has received research support from GlaxoSmithKline and consultant/speaker fees/honoraria from Bristol-Myers Squibb, Monogram Bio, Roche, Abbott, Boehringer Ingelheim, Gilead, GSK, Tibotec, and Virco. MY has received travel bursaries and advisory board/lecture fees from Roche. RK has received honoraria and travel grants from Abbott, Boehringer Ingelheim, Bristol-Myers Squibb, Gilead, GlaxoSmithKline and Roche. MF has received honoraria, travelling scholarships and/or research funding from Abbott, Boehringer Ingelheim, Bristol-Myers Squibb, Gilead, GlaxoSmithKline, Pfizer, Roche and Merck. CC has attended paid advisory boards and received research grants, speaker fees and honoraria from Roche Pharmaceuticals and Trimeris in the past 5 years. PS has obtained grants/research support from Merck, Pfizer, Roche, Theratec and Tibotec, has acted as a consultant for Gilead, GSK, Roche, Tibotec and Virco, and has been on the speaker's bureau and/or received honoraria from BMS, Boehringer Ingelheim, Gilead, GSK, Merck, Monogram, Novartis, Roche, Tibotec. CT has received consultancy/speaker honoraria from Roche.

## Authors' contributions

RH was the academic lead on the project. He provided the conceptual basis for the development of study materials, interpreted data and critically revised the first draft of the manuscript. CKo reviewed and critiqued the drafts of the manuscript. CKa participated in the review of the manuscript. BC participated in drafting, critical review, and revision of the manuscript. CF was involved in the study design, acquisition of data and the review of the manuscript. MY contributed to the multi-disciplinary group that synthesized the data from the study into a cohesive form, gave oral presentations on the study and helped produce the written work. RK contributed to the study design advisory board and reviewed drafts of the manuscript. MF contributed to the study concepts, interpretation of data and provided critical comment on drafts of the manuscript. CC participated in the study design and was involved in manuscript reviews. JS contributed to the study design, critically reviewed and revised the manuscript. PS participated in the interpretation of data, critical review and revisions to the manuscript. VC performed the statistical analysis of the data and contributed to the drafting, critical review and revisions of the manuscript. CT provided substantial contribution to the study concepts, analyzed and interpreted data and assisted in drafting and revising the manuscript. All authors approved the final manuscript.
